# A sensitive genetic screen reveals that BAK1 kinase activity is required for its functions in plant immunity, development and cell death control

**DOI:** 10.1007/s44154-025-00213-0

**Published:** 2025-02-10

**Authors:** Fuhao Cui, Wei Sun, Guangyuan Xu, Meitong Liu, Jinggeng Zhou, Wenxian Sun

**Affiliations:** 1 Frontiers Science Center for Molecular Design Breeding (MOE), Department of Plant Pathology, and the Ministry of Agriculture Key Laboratory of Pest Monitoring and Green Management, China Agricultural University, Beijing, 100193 China; 2 Shanghai Key Laboratory of Plant Molecular Sciences, College of Life Sciences, Shanghai Normal University, Shanghai, 200234 China

**Keywords:** *Arabidopsis*, BRI1-ASSOCIATED RECEPTOR KINASE 1 (BAK1), Plant immunity, Development, Cell death

## Abstract

To dissect the signaling networks that orchestrate immune gene expression, we developed a sensitive genetic screen using an ethyl methanesulfonate (EMS)-mutagenized population of *Arabidopsis* transgenic plants. These plants carry an early immune responsive gene *FRK1* (flg22-induced receptor-like kinase 1) promoter fused with a luciferase (LUC) reporter. A series of mutants with altered *pFRK1::LUC* activity were identified and named as *Arabidopsis g*enes *g*overning *i*mmune gene *e*xpression (*aggie*). The *aggie5* mutant showed reduced *pFRK1::LUC* induction in response to multiple MAMPs treatments. Moreover, the activation of the endogenous *FRK1* gene by MAMPs was also compromised in *aggie5*. Significantly, the bacterial flagellin epitope flg22-induced MAPK activation, ROS production, and immune-related gene expression were reduced in *aggie5*. Map-based cloning coupled with next-generation sequencing revealed that *aggie5* encodes a kinase-inactive BAK1, which is involved in brassinosteroid responses, innate immunity, and cell death control. Consistently, the *aggie5/bak1-16* mutant displayed compromised responses to BR treatment. Furthermore, the *aggie5/bak1-16* mutant exhibited seedling lethality when combined with the mutation of its closest homolog BKK1/SERK4. The data suggest that BAK1 kinase activity is required for its multiple functions in plant immunity, development, and cell death control. The isolation and characterization of these *Aggie* genes will contribute to a better understanding of host immune signaling and provide genetic resources to improve crop resistance.

**Supplementary Information:**

The online version contains supplementary material available at 10.1007/s44154-025-00213-0.

## Introduction

To defend against pathogens, plants utilize cell membrane-localized pattern recognition receptors (PRRs) to perceive microbe- or pathogen-associated molecular patterns (MAMPs/PAMPs) to elicit pattern-triggered immunity (PTI). For instance, two well-studied immune receptor kinases (RKs) FLS2 and EFR, recognize bacterial MAMPs flagellin (or the flagellin epitope flg22) and elongation factor EF-Tu (or the EF-Tu epitope elf18) respectively (Felix et al. 1999; Gomez-Gomez and Boller 2000; Kunze et al. 2004; Zipfel et al. 2006). When flg22 or elf18 is detected, FLS2 and EFR recruit the co-receptor BRI1-associated kinase 1 (BAK1), which was initially found to positively regulate brassinosteroid signaling (Li et al. 2002; Nam and Li 2002), to form an immune complex and trigger downstream immune signaling (Chinchilla et al. 2007; Heese et al. 2007; Schulze et al. 2010; Roux et al. 2011). The following activated defense events include phosphorylation of receptor-like cytoplasmic kinases (RLCKs) (Lu et al. 2010; Zhang et al. 2010), the burst of reactive oxygen species (ROS) controlled by the NADPH oxidase RBOHD, phosphorylation of mitogen-activated protein kinases (MAPKs), phosphorylation of Ca^2+^-dependent protein kinases (CDPKs), and activation of immune-related genes (Boller and Felix 2009; Seybold et al. 2014; Cui et al. 2018; Sun and Zhang et al. 2022). Besides associating with RKs, BAK1 also interacts with receptor-like proteins (RLPs) to enhance immune signaling transduction. For example, upon binding of nlp20 (a conserved 20 amino-acid peptide of most Necrosis and ethylene-inducing peptide 1-like proteins), BAK1 is recruited to RLP23-SOBIR1 (Suppressor of BAK1-Interacting Receptor-like kinase 1) complex to activate downstream immune signaling (Albert et al. 2015). Furthermore, BAK1 also functions together with its closest homolog SERK4 (somatic embryogenesis receptor kinases) in *Arabidopsis* to negatively regulate plant cell death control, as seen in the *bak1/serk4* double mutant which shows a seedling lethal phenotype (Chinchilla et al. 2007; He et al. 2007).

Given that BAK1 plays a pivotal role in the regulation of plant immunity, development, and cell death control, the underlying mechanisms have been extensively studied in recent years. Through a forward genetic screen for elf18-insensitive mutants, a novel *BAK1* allele *bak1-5* was isolated. This allele contains a C408Y mutation in the kinase domain (Schwessinger et al. 2011). Despite the attenuation in FLS2- and EFR-mediated immune signaling, BR signaling and BAK1/SERK4-mediated cell death control remain intact in *bak1-5* (Schwessinger et al. 2011). Interestingly, D122N mutation in the third LRR (leucine-rich repeat) of BAK1’s extracellular domain (bak1^elg^) causes it to gain function in BR signaling while losing function in flagellin signaling. This is likely due to the higher affinity of bak1^elg^ for BRI1 and lower affinity for FLS2 (Jaillais et al. 2011). In another high-throughput genetic screen for components involved in the regulation of flg22-triggered Ca^2+^-dependent signaling, ten novel *bak1* alleles were identified. These alleles were tentatively named from *bak1-6* to *bak1-15*, all of which exhibited attenuated flg22-mediated seedling growth inhibition (SGI) phenotype (Ranf et al. 2012). Regarding BAK1/SERK4-mediated cell death control, it has been reported that N-glycosylation is essential for activating *bak1*/*serk4* cell death, and CRK4 (cysteine-rich receptor-like kinase 4) is likely one of the glycosylated proteins (de Oliveira et al. 2016). Moreover, BAK1/SERK4-mediated phosphorylation regulates the stability of Ca^2+^ channel CNGC20/CNGC19 precisely control *bak1*/*serk4* cell death (Yu et al. 2019). Recently, Yu et al. found that *Arabidopsis* receptor kinase BTL2 (BAK-TO-LIFE 2) serves as a surveillance rheostat sensing the perturbation of BAK1/SERK4 immune co-receptors to promote NLR-mediated phytocytokine signaling, ensuring plant immunity (Yu et al. 2023).

In *Arabidopsis*, not much is known about the kinase activity of BAK1 in relation to plant immunity, development, and cell death control. Here, through a forward genetic screen for *aggie* (*A*rabidopsis *g*enes *g*overning *i*mmune gene *e*xpression) mutants involved in controlling plant immune signaling, we identified a novel BAK1 allele, *bak1-16*, which has a D416N mutation in the kinase domain and exhibits reduced immune responses, BR signaling, and BAK1/SERK4-mediated cell death control.

## Results

### The *aggie5* mutant shows decreased *pFRK1::LUC* activity to several MAMPs

**Fig. 1:**
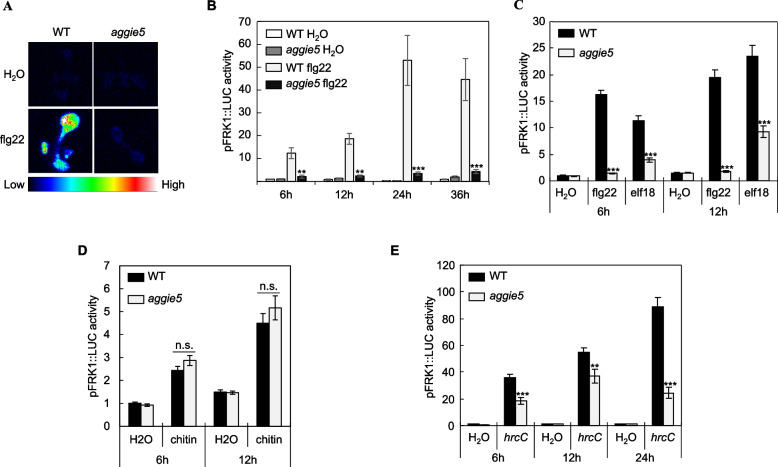
Decreased *pFRK1::LUC* activity triggered by MAMPs and *Pst hrcC* strain. **A** Luciferase activity in 10-day-old *pFRK1::LUC*, and *aggie5* seedlings. Seedlings were treated with or without 10 nM flg22 for 12 h and then photographed with an EMCCD camera. **B** Time course of *pFRK1::LUC* activity. The seedlings were treated with 10 nM flg22 over 36 h. The data are shown as means ± standard error (SE) from at least 6 seedlings for each time point. Asterisks denote significant differences compared to WT. Asterisks indicate significant differences compared to WT (Student's *t*-test; **P < 0.01, ***P < 0.001). **C** The *pFRK1::LUC* activity triggered by different MAMPs. Ten-day-old seedlings were treated with 10 nM flg22 or elf18 for 6 h and 12 h. The data are shown as means ± SE from at least 6 seedlings for each time point. Asterisks indicate significant differences compared to WT (Student's *t*-test; ***P < 0.001). **D** The *pFRK1::LUC* activity triggered by fungal chitin. Ten-day-old seedlings were treated with 50 μg/ml chitin for 6 h and 12 h. The data are shown as means ± SE from at least 6 seedlings for each time point. “n.s.” indicates no significant differences compared to WT (Student's* t*-test). **E** The *pFRK1::LUC* activity triggered by *Pst hrcC* strain. Ten-day-old seedlings were treated with *Pst hrcC* at an optical density at 600 nm (OD_600_) of 0.1 over 24 h. The data are shown as means ± SE from at least 6 leaves for each time point. Asterisks indicate significant differences compared to WT (Student's *t*-test; **P < 0.01, ***P < 0.001). These experiments were repeated 3 times with similar results

To identify the components that regulate immune gene expression in plants, we established a sensitive genetic screen system. We used an EMS-mutagenized population of *Arabidopsis* transgenic plants, which contain a luciferase reporter gene under the control of the *FRK1* (flg22-induced receptor-like kinase 1) promoter (*pFRK1::LUC*). The activity of *pFRK1::LUC* is highly induced by multiple MAMPs (Asai et al. 2002; He et al. 2006). Theoretically, the strength of plant immune signaling is positively correlated with the *pFRK1::LUC* activity upon MAMPs treatment. A series of mutants with significant changes in *pFRK1::LUC* activity, which is induced by flg22 treatment or inoculation with *Pseudomonas syringae* pv *tomato* (*Pst*) DC3000 type III secretion system mutant *hrcC*, were identified and named as *Arabidopsis* genes governing immune gene expression (*aggie*) (Feng et al. 2015; Li et al. 2014; Wei et al. 2023). The *aggie5* mutant displays reduced *pFRK1::LUC* activity after flg22 treatment compared to wild-type (WT) *pFRK1::LUC* transgenic seedlings (Fig. 1A, 1B and 1C). The decreased *pFRK1::LUC* activity in *aggie5* was observed throughout a 36-h time course period with flg22 treatment (Fig. 1B). Similarly, elf18-induced *pFRK1::LUC* activity was also significantly reduced in *aggie5* (Fig. 1C), indicating that Aggie5 functions as a convergent component downstream of FLS2 and EFR. Interestingly, the fungal chitin-triggered *pFRK1::LUC* activity in *aggie5* was comparable to that in the WT (Fig. 1D), suggesting that Aggie5 functions either independently or downstream of chitin receptors.

Given that *hrcC* is the nonpathogenic strain of *Pseudomonas syringae* pv. *tomato* (*Pst*) DC3000, which lacks the ability to deliver bacterial type III effector proteins into plant cells, it could be regarded as a mixture of bacterial MAMPs. We discovered that *aggie5* seedlings displayed attenuated *pFRK1::LUC* activity when treated with *hrcC* in comparison to the WT (Fig. 1E). All in all, the results suggest that Aggie5 positively regulates the flg22- and elf18-mediated immune signaling pathways but does not influence the chitin signaling pathway.

### Flg22-triggered immune responses are diminished in *aggie5*

**Fig. 2:**
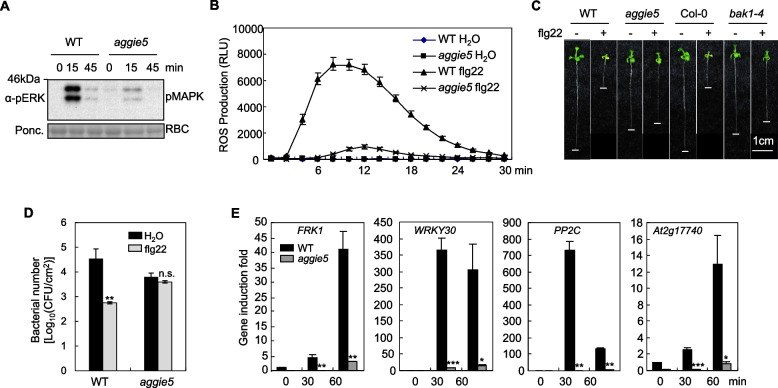
Reduced flg22-induced immune signaling in *aggie5*. **A** Flg22-induced MAPK activation. Twelve-day-old seedlings were treated with 100 nM flg22 for different time points. MAPK activation was analyzed using an α-pERK antibody (top panel), and protein loading was shown by Ponceau S (bottom panel) staining of RuBisCO (bottom panel). **B** Flg22 triggered ROS burst. Leaf discs were treated with H_2_O or 100 nM flg22 over 30 min. The data are shown as means ± SE from 12 leaf discs. **C** *bak1-16* mutants show reduced sensitivity to flg22 in seedling growth inhibition assay. Col-0, FRK1-LUC, *bak1-4*, and *bak1-16* seedlings were grown for 10 days in presence of 400 nM flg22. **D** *bak1-16* mutants are compromised in flg22-triggered immunity to *Pst* DC3000. Results of a bacterial growth assay of 4-week-old FRK1-LUC and *bak1-16* plants, 4 days after infection, are shown. The data are shown as means ± SE from three biological replicates. Asterisks indicate significant differences compared to corresponding values of H_2_O treated leaves (Student's *t*-test; **P < 0.01, n.s. indicates no significant difference). **E** Endogenous expression of *FRK1*, *WRKY30*, *PP2C*, and *At2g17740*. Twelve-day-old seedlings were treated with 100 nM flg22 for qRT-PCR analysis. The data are shown as means ± SE from three biological replicates. Asterisks indicate significant differences compared to WT (Student's t-test; **P < 0.05, **P < 0.01, ***P < 0.001). These experiments were repeated 3 times with similar results

In order to explore whether other early immune responses are impaired in *aggie5*, we further examined flg22-triggered ROS production, MAPK phosphorylation, and immune-related gene expression in *aggie5*. We found that flg22-induced ROS production was significantly reduced in *aggie5* adult plants (Fig. 2A). Next, we detected flg22-triggered MAPKs activation using an α-pERK antibody in WT and *aggie5* mutant seedlings. Flg22-activated MPK3, MPK6, and MPK4/11 phosphorylation was considerably reduced in *aggie5* compared to WT. These results imply that Aggie5 functions either dependently or upstream of ROS production and MAPKs phosphorylation in the FLS2 signaling pathway. Furthermore, we tested the SGI (seedling growth inhibition) phenotype of *aggie5* caused by flg22, which serves as an assay for determing plant sensitivity to MAMPs (Gomez-Gomez et al. 1999). In accordance with the reduced *pFRK1::LUC* activity triggered by flg22 (Fig. 1A and 1B), the *aggie5* seedlings displayed insensitivity in the SGI triggered by flg22 (Fig. 2C). This is reminiscent of the *bak1-4* null mutant, which has impaired flg22-triggered SGI and was thus used as a negative control in this context (Chinchilla et al. 2007).

Since *aggie5* was impaired in flg22 signaling (Fig. 1A to 1C), we hypothesized that *aggie5* might also be impaired in flg22-induced protection against bacterial pathogen infection. Indeed, bacterial pathogen multiplication was not restricted when *aggie5* was pretreated with flg22 before hand-inoculation with *Pst* DC3000, compared to the WT (Fig. 2D). Intriguingly, we observed that *aggie5* was significantly more resistant to *Pst* DC3000 when plant leaves were pretreated with H_2_O, suggesting that Aggie5 mutation may activate plant basal resistance to bacterial pathogens.

Although MAMPs-induced *pFRK1::LUC* activity was decreased in *aggie5*, it still remains to be determined whether MAMP-induced endogenous *FRK1* gene expression level was also decreased. Therefore, we quantified the flg22-induced endogenous *FRK1* gene expression using quantitative real-time PCR (qRT-PCR). Consistent with the *pFRK1::LUC* expression pattern, the flg22-induced endogenous *FRK1* gene expression level in *aggie5* seedlings was considerably lower compared with that in the WT (Fig. 2E). Besides, we also examined the expression level of several other MAMP-responsive genes. In line with the endogenous *FRK1* gene induction pattern, the expression of all three other genes, namely *WRKY30*, *PP2C*, and *At2g17740* was also significantly lower compared with the WT (Fig. 2E). Taken together, these results suggest that Aggie5 plays a positive role in the FLS2 immune signaling pathway.

### *Aggie5* encodes a BAK1 kinase-inactive mutant

**Fig. 3:**
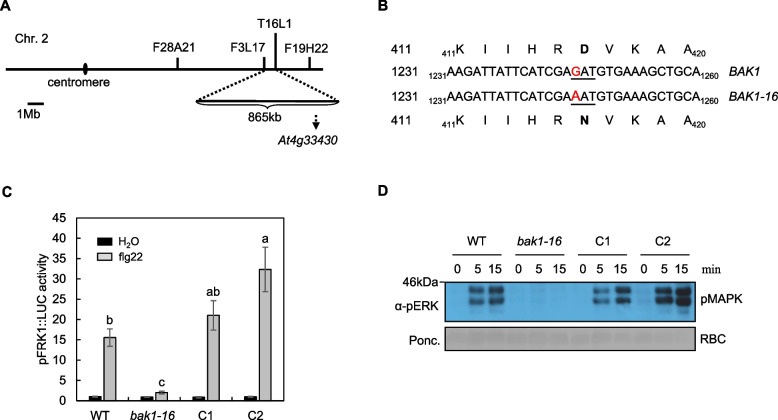
*bak1-16* encodes a BAK1 kinase-inactive mutant. **A** Map position of *bak1-16* on the long arm of Chromosome 4 based on linkage analysis using segregating F2 progeny from *bak1-16* crossed to Ler. **B** Position of *bak1-16* mutation, indicating both genomic and peptide transitions. **C** Flg22-induced *pFRK1::LUC* activity is complemented by *pBAK1::BAK1* transformation in *bak1-16*. C1, and C2 are two independent transgenic lines of *aggie5* complemented with *pBAK1::BAK1*. Ten-day-old seedlings were treated with 100 nM flg22 for 12 h. The data are shown as means ± SE from at least 10 seedlings. The different lower case letters indicate a significant difference (*P* < 0.05) according to a one-way ANOVA with Tukey’s test. **D** Flg22-induced MAPK activation. Ten-day old seedlings were treated with 100 nM flg22 for different time points. MAPK activation was analyzed using an α-pERK antibody (top panel), and protein loading was shown by Ponceau S (bottom panel) staining for RuBisCO (RBC). These experiments were repeated 3 times with similar results

To identify the causative mutation in *aggie5*, we crossed *aggie5* (in the Col-0 accession background) to the Ler accession. By utilizing a map-based cloning method, we narrowed down the corresponding mutation to Chromosome 4, located between markers F3L17 and T16L1, which are 859 kilobase pairs apart (Fig. 3A). When combined with next-generation sequencing data of the whole genomic DNAs of *aggie5* and *pFRK1::LUC* transgenic plants, the comparative sequence analysis identified a G to A mutation at the 1246 bp position in the 10th exon of *At4g33430* (Fig. 3B). The mutation in *aggie5* was further verified by Sanger sequencing of *At4g33430*. *At4g33430* encodes BAK1, and the mutation in the *aggie5* mutant results in an amino acid change from Aspartic acid (D) at position 416 to Asparagine (N) in the catalytic loop domain (Fig. 3B). Besides, there is another G to A nucleotide mutation at the 642 bp position in the 7th exon of *BAK1*. However, this nucleotide change is a non-sense mutation that does not impact BAK1 protein-coding. The catalytic site D416 has been reported to play a central role in BAK1 kinase activity in vitro (Schwessinger et al. 2011; Yan et al. 2012; Zhang et al. 2021). Nevertheless, there is no report regarding D416’s role in BAK1 kinase activity in vivo. We thus tentatively rename *aggie5* as *bak1-16*, following previously reported *bak1* mutant alleles named from *bak1-1* to *bak1-15* (Kemmerling et al. 2007; Ranf et al. 2012).

To confirm that the D416N amino acid change in BAK1 is the causative mutation responsible for the decreased flg22-triggered *pFRK1::LUC* activity and other immune-related responses, a construct containing the full-length genomic DNA of *BAK1* under the control of its own promoter (1651 bp) was introduced into *aggie5* through *Agrobacterium*-mediated transformation. The *pFRK1::LUC* activity induced by flg22 was restored to WT level in two independent complementation lines, C1 and C2, indicating that BAK1 is the mutated gene responsible for decreased *pFRK1::LUC* induction triggered by flg22 (Fig. 3C). Moreover, flg22-induced MAPK activation was also restored to the same level as the WT (Fig. 3D). These results genetically imply that BAK1 kinase activity is essential for the flg22-mediated signaling pathway.

### *bak1-16* is impaired in brassinosteroid signaling

**Fig. 4:**
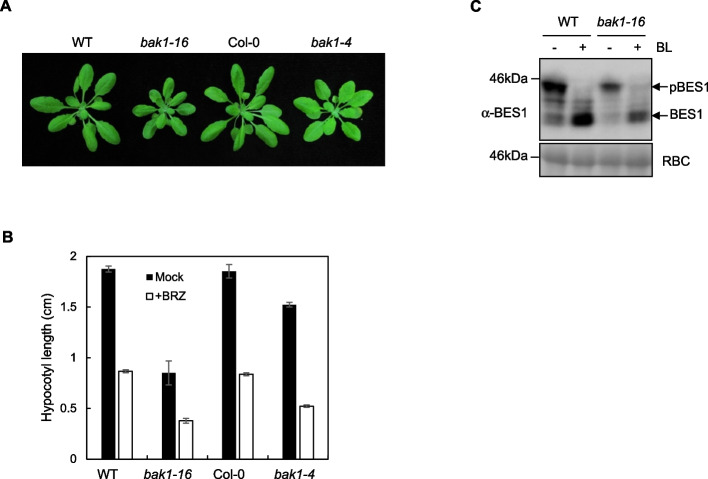
*bak1-16* is impaired in BR signaling. **A** Morphology of FRK1-LUC, Col-0, *bak1-16*, and *bak1-4* plants. The pictures were taken when plants were grown in soil for 4 weeks. **B** The hypocotyl length of 6-day-old dark-grown seedlings in the absence or presence of 2 μM BRZ. Data are presented as mean ± SE (*n* = 14, 9, 13, 27 for absence of BRZ, and *n* = 14, 10, 13, 29 for presence of 2 μM BRZ respectively). **C** BL treatment induces the de-phosphorylation of BES1 in the *bak1-16* mutant. Ten-day-old seedlings of FRK1-LUC and *bak1-16* were treated with 0 or 1 µM BL for 3 h. Total proteins were analyzed by an immuo-blotting assay using a specific α-BES1 antibody (top panel). The protein loading is shown by Ponceau S (bottom panel) staining for RBC. These experiments were repeated 2 times with similar results

Previous study reported that *bak1* loss-of-function alleles display a semi-dwarf cabbage-like phenotype similar to the *bri1* null mutant (Li et al. 2002; Nam and Li 2002). Thus we further tested whether *bak1-16* was impaired in BR signaling. Apparently, *bak1-16* mutant plants exhibited round leaves and very short petioles, similar to *bak1-4* (Fig. 4A). Since plant morphology may not always correlate with defects in BR responses, we further investigated the effect of BR biosynthetic inhibitor brassinazole (BRZ) on *bak1-16* seedlings. Consistent with a previous report (Lin et al. 2013), without BRZ treatment, the hypocotyl length of *bak1-4* was shorter than the wild type Col-0 when seedlings were grown vertically in darkness (Fig. 4B). Interestingly, the hypocotyl length of *bak1-16* was shorter than that of *bak1-4*, while the hypocotyl length was comparable between the WT (FRK1-LUC) and Col-0 seedlings (Fig. 4B). In the presence of brassinazole (BRZ), an inhibitor of BR biosynthesis, the hypocotyl length of *bak1-16* seedlings was further shorter than that of *bak1-4* (Fig. 4B). As expected, both of the hypocotyl elongation of *bak1-16* and *bak1-4* mutants were shorter than that of WT and Col-0 seedlings (Fig. 4B). As a hallmark of active BRI1 signaling, the downstream transcription factor BES1 (*bri1*-Ems-Suppressor 1) is de-phosphorylated to positively regulate the expression of BR responsive genes (Yin et al. 2002). We examined the endogenous BES1 proteins’ phosphorylation status using a specific α-BES1 antibody. In WT seedlings, BL induced BES1 de-phosphorylation, as indicated by the mobility shift of BES1 proteins from high molecular weight to low molecular weight in a Western blot (Fig. 4C). Compared with WT seedlings, the *bak1-16* mutant exhibited a decreased amount of both phosphorylated and de-phosphorylated BES1 proteins, either with or without BL treatment (Fig. 4C). This is consistent with the shorter hypocotyls of *bak1-16* seedlings compared to those of WT (Fig. 4B). Taken together, these results suggest that BAK1 kinase activity is essential for the BR signaling pathway.

### *bak1-16* is impaired in cell death control

**Fig. 5:**
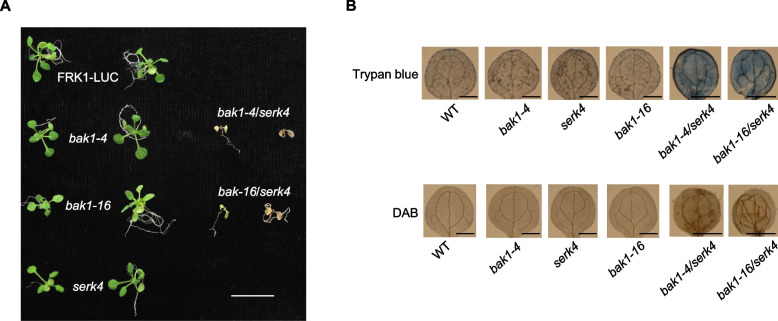
Cell death control is compromised in *bak1-6*. **A** Picture of representative individuals of 2-week-old seedlings of FRK1-LUC, *bak1-4*, *bak1-16*, *serk4*, *bak1-4*/*serk4,* and *bak1-16*/*serk4* mutants. Scale bar represents 2 cm. **B** Trypan blue staining (upper panel) and DAB staining (lower panel) of true leaves of FRK1-LUC, *bak1-4*, *bak1-16*, *serk4*, *bak1-4*/*serk4* and *bak1-16*/*serk4* mutants. Scale bar represents 0.2 cm. These experiments were repeated 2 times with similar results

Since BAK1 functions together with BKK1 in cell death control (Chinchilla et al. 2007; He et al. 2007), we further tested whether *bak1-16* is impaired in cell death control in the *bkk1* background. We crossed *bak1-16* with the null mutant *bkk1-1*. *bak1-16/bkk1-1* double mutant seedlings exhibit severe spontaneous cell death and constitutive H_2_O_2_ accumulation in cotyledons even when grown in a sterile environment (Fig. 5A and 5B). In contrast, *bak1-4/bkk1-1* double mutant displays similar cell death and elevated H_2_O_2_ level, which is consistent with a previous report (He et al. 2007). Consistently, other BAK1 kinase-inactive variants have been proved to be critical for cell death control in *Arabidopsis*, as the lethality of *bak1-4*/*bkk1-1* seedling cannot be rescued by either BAK1 K317E, S286D, or T455A (Wang et al. 2008). The result indicates that BAK1 kinase activity is required for cell death control in *Arabidopsis* seedlings.

## Discussion

To identify novel plant immune signaling regulators, we established a sensitive forward genetic screen system by generating an EMS-mutagenized pool based on *pFRK1::LUC* transgenic *Arabidopsis*. Through this system, a series of mutants with altered flg22-triggered *pFRK1::LUC* activity were isolated, and the function of novel plant immune regulators were characterized (Li et al. 2014; Feng et al. 2015; Wei et al. 2023). In this study, we identified the kinase-inactive variant *bak1-16* as the target gene responsible for the decreased flg22-triggered *pFRK1::LUC* activity in the *aggie5* mutant. BAK1 is a plasma membrane-resident co-receptor kinase that mediates multiple PRR signaling pathways (Couto and Zipfel 2016). We found that BAK1 kinase activity is essential for its function in plant immune signaling, development, and cell death control.

Although previous studies have demonstrated that *bak1-16* loses kinase activity in vitro and that BAK1 kinase activity is required for SOBIR1-mediated immunity in *Nicotiana benthamiana* (Schwessinger et al. 2011; van der Burgh et al. 2019), no in vivo experimental data have verified that the kinase activity of BAK1 is required in plant innate immunity in *Arabidopsis*. Here, we found flg22- and elf18-triggered immune signaling was significantly compromised in *bak1-16* (Figs. 1 and 2). The strongly impaired immune signaling in *bak1-16* is not caused by the binding affinity change of BAK^D416N^ to FLS2 or EFR. This is because it was previously revealed that flg22- and elf18-induced heteromerization of FLS2 and EFR with BAK1^D416N^ is unaltered compared with wild-type BAK1 (Schwessinger et al. 2011). Based on the impaired immune responses in *bak1-16* (Fig. 2A, 2B and 2E), *bak1-16* is believed to be more susceptible to bacterial infection. However, surprisingly, *bak1-16* is significantly more resistant to *Pst* DC3000 infection than WT when pre-treated with H_2_O (Fig. 2D). Yamada et al. reported that *bak1-4* is more resistant to *Pst* DC3000 (Yamada et al. 2016), most likely because BAK1 protein depletion activates ADR1-mediated nucleotide-binding leucine-rich repeat protein (NLR)-mediated signaling (Wu et al. 2020). It is conceivable that similar to BAK1 depletion, the kinase-dead BAK1^D416N^ variant could also be recognized as a modified guardee to trigger NLRs activation to restrict *Pst* DC3000 multiplication (Wu et al. 2020).

BR signaling is more severely impaired in *bak1-16* mutant compared to the *bak1-4* knockout mutant (Fig. 4). In *bak1-4*, although BAK1 protein is depleted, BAK1 paralogs may still function in BR signaling (Meng et al. 2015). In contrast, the kinase-dead BAK1-16 protein exists and may interfere with the function of its paralogs in mediating BR signaling. The molecular mechanism remains to be determined. Although BR signaling is not impaired in *bak1-5*, in which BAK1-5 (BAK1^C408Y^) is a hypoactive kinase (Schwessinger et al. 2011), other amino acids critical for BAK1 kinase activity are required to rescue the growth phenotype of the weak BR-insensitive mutant *bri1-5* (Wang et al. 2008).

Compared with the T-DNA knockout mutants of *bak1-3* and *bak1-4*, in which BAK1 protein is depleted, the full-length of the kinase-dead BAK1-16 protein still exists in *bak1-16*. Thus, our *bak1-16* allele is an important plant genetic material for studying BAK1 kinase activity in PRRs-mediated signaling pathways. We are deploying next-generation sequencing coupled with map-based cloning to uncover the gene identities of other *aggie* mutants. The isolation and characterization of these *Aggie* genes will help understand host immune signaling and provide genetic resources to improve crop resistance.

## Conclusions

In summary, we identified a novel BAK1 allele through a sensitive high-throughput genetic screen for *Arabidopsis g*enes *g*overning *i*mmune gene *e*xpression (*aggie*). The *aggie5* mutant showed reduced MAMP-triggered *pFRK1::LUC* induction and endogenous *FRK1* gene activation. Significantly, flg22-induced MAPK activation, ROS production, and immune-related genes expression were decreased in *aggie5*. Map-based cloning combined with next-generation sequencing revealed that *aggie5* encodes a kinase-dead BAK1, which is involved in development, innate immunity, and cell death control. Consistently, the *aggie5/bak1-16* mutant showed impaired responses to BR treatment. Moreover, the *aggie5/bak1-16* mutant exhibited seedling lethality when combined with the mutation of its closest homolog BKK1/SERK4. The data indicate that BAK1 kinase activity is crutial for its multiple functions in plant immunity, development, and cell death control.

## Materials and methods

### Plant growth conditions, and pathogen inoculation

*Arabidopsis* accession Col-0, Ler, *pFRK1::LUC* transgenic plants, *aggie5* mutant, *bak1-4* (SALK_116202) and *bkk1-1* (SALK_057955) were grown in soil (Metro Mix 366) with 60% humidity, 75 μE m^−2^ s^−1^ light and a 12-h photoperiod at 23℃. Four-week-old plants were used for protoplast isolation and bacterial infiltration. To detect MAPK activation and gene induction, 10-day-old seedlings grown on ½ solid Murashige-Skoog agar medium were transferred to double distilled water for overnight and then treated with 100 nM flg22 for the indicated time points.

### Quantitative RT-PCR (qRT-PCR) analysis

Total RNA of 10-d-old *Arabidopsis* seedlings treated with the indicated peptides was extracted using TRIzol reagent (Invitrogen). Total RNA was reverse-transcribed using an oligo(dT) primer and reverse transcriptase. The qRT-PCR analysis was performed using the iTaq SYBR green Supermix (Bio-Rad) with an ABI GeneAmp PCR System 9700. The expression of each gene was normalized to the expression of *UBQ10*.

### MAPK activation assay

Ten-day-old *Arabidopsis* seedlings were transferred from solid ½ Murashige-Skoog agar medium to ddH_2_O overnight. The ddH_2_O was replaced with fresh ddH_2_O, and flg22 was applied for 0, 15, 45 min or 0, 5, 15 min. Seedlings were immediately frozen in liquid nitrogen. Then the total protein was extracted in twofold concentrated SDS loading buffer and boiled at 95℃ for 10 min.

### Seedling growth inhibition assay

Three-day-old seedlings grown on vertical solid ½ MS medium were transferred into a 24-well tissue culture plate either with or without 400 nM flg22, then seedlings were grown for one week. Pictures were taken 7 d later.

### Trypan blue and DAB staining

Trypan blue staining and 3,3′-diaminobenzidine (DAB) staining were performed according to procedures described previously with modifications. Briefly, the excised plant tissues were immersed in either trypan blue staining solution (2.5 mg ml^–1^ trypan blue in lactophenol (lactic acid, glycerol, liquid phenol and H_2_O in a ratio of 1:1:1:1)) or DAB solution (1 mg ml^–1^ DAB in 10 mM Na_2_HPO_4_ and 0.05% Tween 20). The samples were subjected to vacuum infiltration for 30 min and then incubated for 8 h at 25 °C with gentle shaking at a speed of 75 rpm. Subsequently, the samples were transferred to trypan blue destaining solution (ethanol and lactophenol in a ratio of 2:1) or DAB destaining solution (ethanol, acetic acid and glycerol in a ratio of 3:1:1) and incubated at 65 °C for 30 min. Finally, the samples were incubated in fresh destaining solution at room temperature until they were complete destained. Pictures were taken under a dissecting microscope with the samples placed in 10% glycerol.

## Supplementary Information

Supplementary Material 1.

## Data Availability

For agrobacterium strains, plasmids, or *Arabidopsis* genotypes generated in this study, please contact the corresponding author who will make it available upon reasonable request.

## Abbreviations

BAK1: BRI1-Associated Receptor Kinase 1
BES1: BRI1-Ethyl methyl sulfon-SUPPRESSOR 1
BKK1: BAK1-LIKE 1
BL: Brassinolide
BR: Brassinosteroid
BRI1: BRASSINOSTEROID INSENSITIVE 1
BRZ: Brassinazole
CDPK: Calcium-Dependent Protein Kinase
CNGC: Cyclic nucleotide-gated ion channel
CRK: Cysteine-rich receptor-like kinase
EFR: EF-TU RECEPTOR
EF-Tu: Elongation factor thermo-unstable
elf18: The first 18 amino acids of the N-terminus of EF-Tu
EMS: Ethyl methanesulfonate
flg22: The first 22-amino acid peptide of bacterial flagellin
FLS2: FLAGELLIN SENSING 2
FRK1: FLG22-INDUCED RECEPTOR-LIKE KINASE1
LUC: Luciferase
MAMP: Microbial associated molecular patterns
MAPK: Mitogen-activated protein kinase
NADPH: Educed form of nicotinamide adenine dinucleotide phosphate
nlp: A necrosis- and ethylene-inducing peptide 1 (Nep1)-like protein
PAMP: Pathogen-associated molecular patterns
PP2C: Protein phosphatase 2C
PRR: Pattern recognition receptors
*Pst*: *Pseudomonas syringae* Pv *tomato*
PTI: Pattern-triggered immunity
RBOHD: RESPIRATORY BURST OXIDASE HOMOLOG D
RBC: RuBisCO
RK: Receptor kinase
RLCK: Receptor-like cytoplasmic kinases
RLP: Receptor-like protein
ROS: Reactive oxygen species burst
SERK: SOMATIC EMBRYOGENESIS RECEPTOR-LIKE KINASE
SOBIR1: SUPPRESSOR OF BIR1
WT: Wild-type
