# Supplementary material for: A sensitive genetic screen reveals that BAK1 kinase activity is required for its functions in plant immunity, development and cell death control
Source: Stress Biol. 2025 Feb 10;5(1):13. doi: 10.1007/s44154-025-00213-0 (PMC11807947; doi:10.1007/s44154-025-00213-0)
Supplement: Supplementary file 1 — Supplementary Material 1. [file 44154_2025_213_MOESM1_ESM.docx]

| Primers used in this study | | |
| --- | --- | --- |
| Primer name | Forward primer and reverse primers (5’ to 3’) | Purpose |
| F1K23-1F | TGTGGCATAATTTTGAATCATCA | For rough mapping on chromosome 1 |
| F1K23-1R | GTCCACTGCCCAGTCTAGGA | For rough mapping on chromosome 1 |
| F7F22-1F | AAGCTCCAATCCAGAGGAGTC | For rough mapping on chromosome 1 |
| F7F22-1R | CATCCTCGTCCATCAGCTCT | For rough mapping on chromosome 1 |
| F23H11-F | GATATGGGAGTAAGTATGAAATCGG | For rough mapping on chromosome 1 |
| F23H11-R | TTCGTCCGGGTAAAAGTCAAG | For rough mapping on chromosome 1 |
| F9O13-1F | TTGTATCATCAAAGGTTCCTGG | For rough mapping on chromosome 2 |
| F9O13-1R | AAATTCCGTCTTCGTATCGTCT | For rough mapping on chromosome 2 |
| F25I18-F | CGTATACTAGCATATCTGTG | For rough mapping on chromosome 2 |
| F25I18-R | AAGGACTTCCCGTTCTTGGT | For rough mapping on chromosome 2 |
| F2H17-F | ATTGCATACCACGCAGTTCAC | For rough mapping on chromosome 2 |
| F2H17-R | CCATTTTGCCCTTTCCTTCTAC | For rough mapping on chromosome 2 |
| MDC16-1F | TTTGGCTCGGACAAGGTTT | For rough mapping on chromosome 3 |
| MDC16-1R | CGTTGTAATCGGGAAAATGCT | For rough mapping on chromosome 3 |
| K16N12-1F | AGAGGAAATGTGGTGATGTAGC | For rough mapping on chromosome 3 |
| K16N12-1R | TCATCATTCTCATCCAAACCC | For rough mapping on chromosome 3 |
| T6H20-1F | CGGCTGAAACTTGGAAGGGAC | For rough mapping on chromosome 3 |
| T6H20-1R | AGGAAGAACGTGTGATTGTG | For rough mapping on chromosome 3 |
| F19H22-1F | CCGGCTGGAACTTGAGACTA | For rough mapping on chromosome 4 |
| F19H22-1R | CTTTACAAACCCGGAAGCAG | For rough mapping on chromosome 4 |
| F28A21-1F | GCATCATCATTCATCACCAAC | For rough mapping on chromosome 4 |
| F28A21-1R | TGTGAAGTGTTTGTCTTTGTG | For rough mapping on chromosome 4 |
| C6L9-1F | TCGTCGTTCTTTTCGGCTTTC | For rough mapping on chromosome 4 |
| C6L9-1R | CGGACTTATTGGTGCTTTTGGTA | For rough mapping on chromosome 4 |
| MHF15-1F | CTCCTCCTTTAATTTTCTCTCTGTG | For rough mapping on chromosome 5 |
| MHF15-1R | AGTTCCAGCTTTGGACTTCTTC | For rough mapping on chromosome 5 |
| MPL12-1F | GTCCCCAAAACCAATCATAAG | For rough mapping on chromosome 5 |
| MPL12-1R | TCCGAGTGAGAAGAGAGTTTG | For rough mapping on chromosome 5 |
| MNC17-1F | GTACCGGATCTGTGTTGTGAAG | For rough mapping on chromosome 5 |
| MNC17-1R | GTGCTCAAGGAAATGGGATAG | For rough mapping on chromosome 5 |
| F1C12-1F | TCTGGAGCCAAAATTAAAAGTC | For fine mapping on chromosome 4 |
| F1C12-1R | GTCCACCGATTTAGAGAGTAAAAG | For fine mapping on chromosome 4 |
| F1N20-1F | GTCAATCCAATACCGCCAATAC | For fine mapping on chromosome 4 |
| F1N20-1R | CTCCATTAGCCAACACATCATAGT | For fine mapping on chromosome 4 |
| F7K2-1F | GATTTACGGCGGTTCTTGATG | For fine mapping on chromosome 4 |
| F7K2-1R | GCACCACACACATTCTCCTCA | For fine mapping on chromosome 4 |
| F6I7-1F | GTGTGTGGTTTTTACGCTTGTTT | For fine mapping on chromosome 4 |
| F6I7-1R | AAAAGATTGGGCTGTTGATGG | For fine mapping on chromosome 4 |
| F27B13-1F | TGCTGATACTTGCTTTCGCTTTG | For fine mapping on chromosome 4 |
| F27B13-1R | GCCTTTTGCTCTCTGTTTTCTGG | For fine mapping on chromosome 4 |
| F3L17-1F | ACGTCTAAACATGAACCAAGC | For fine mapping on chromosome 4 |
| F3L17-1R | CCACTTATCTTGATGTTGGGA | For fine mapping on chromosome 4 |
| T16L1-1F | TCTTCTGGAGCTTGGATAAAGG | For fine mapping on chromosome 4 |
| T16L1-1R | CAACAACAGCTCTTACCACGTT | For fine mapping on chromosome 4 |
| F28A23F | CACGTGGCAAAAAGTATCCA | For fine mapping on chromosome 4 |
| F28A23R | GGAGCCAAGATTTTCACACAA | For fine mapping on chromosome 4 |
| F23E13-1F | TCGCTAACCCTCTCACGAA | For fine mapping on chromosome 4 |
| F23E13-1R | TGGCTGTGAGTGAGTGAAG | For fine mapping on chromosome 4 |
| UBQ10-qRT-F | AGATCCAGGACAAGGAAGGTATTC | For qRT-PCR |
| UBQ10-qRT-R | CGCAGGACCAAGTGAAGAGTAG | For qRT-PCR |
| FRK1-qRT-F | ATCTTCGCTTGGAGCTTCTC | For qRT-PCR |
| FRK1-qRT-R | TGCAGCGCAAGGACTAGAG | For qRT-PCR |
| WRKY30-qRT-F | GCAGCTTGAGAGCAAGAATG | For qRT-PCR |
| WRKY30-qRT-R | AGCCAAATTTCCAAGAGGAT | For qRT-PCR |
| PP2C-qRT-F | CGTGTTGGGGATTGATTCG | For qRT-PCR |
| PP2C-qRT-R | AGAGCTCGGGCGGTTATG | For qRT-PCR |
| At2g17740-qRT-F | TGCTCCATCTCTCTTTGTGC | For qRT-PCR |
| At2g17740-qRT-R | ATGCGTTGCTGAAGAAGAGG | For qRT-PCR |
| p1300-pBAK1-BAK1-F | TACAAATCTATCTCTGGTACCATGGGCTGCTTTCACT | For pCABIA1300-pBAK1-BAK1 |
| p1300-pBAK1-BAK1-R | AACGTCGTATGGGTAAGGCCTTTATCTTGGACCCGAGGG | For pCABIA1300-pBAK1-BAK1 |
| BAK1-F1 | ATGGAACGAAGATTAATGATC | For BAK1-16 cDNA sequencing |
| BAK1-F2 | GCGAAGGAAAAAGCCGCAGGAC | For BAK1-16 cDNA sequencing |
| BAK1-R1 | CCACCAAGCTAGTGCAA | For BAK1-16 cDNA sequencing |
| BAK1-R2 | TCTTGGACCCGAGGGGTATTC | For BAK1-16 cDNA sequencing |
